# Physicochemical, Stress Degradation Evaluation and Pharmacokinetic Study of AZGH102, a New Synthesized COX2 Inhibitors after I.V. and Oral Administration in Male and Female Rats.

**Published:** 2017

**Authors:** Hoda Bahmanof, Simin Dadashzadeh, Afshin Zarghi, Alireza Shafaati, Seyed Mohsen Foroutan

**Affiliations:** a *Department of Pharmaceutics, School of Pharmacy & Protein Technology Research Center, Shahid Beheshti University of Medical Sciences, Tehran, Iran.*; b *Department of Medicinal Chemistry, School of Pharmacy, Shahid Beheshti University of Medical Sciences, Tehran, Iran.*

**Keywords:** Pharmacokinetic, Sex-dependent, Ketoprofen, Selective COX-2 inhibitors, AZGH 102

## Abstract

Coxibs such as celecoxib, rofecoxib, and valdecoxib are introduced as selective COX-2 inhibitors to the market. It has been reported that inhibition of COX-2 beside traditional effects of NSAIDs, reduces the risk of colorectal, breast and lung cancers and also slow the progress of Alzheimer’s disease. Zarghi et al. reported 8-benzoyl-2-(4-(methylsulfonyl)phenyl)quinoline-4-carboxylic acid (AZGH 102) as a novel compound with similar IC50 to celecoxib besides improved selectivity index (COX-1/COX-2 inhibitory potency) in comparison with celecoxib.

In this study, the physicochemical properties of AZGH 102 such as solubility, log P, and stability were evaluated and the pharmacokinetic characteristics of this compound following intravenous (10 mg/Kg), and oral administration (20 mg/Kg), to male and female Wistar rats were investigated.

As the data demonstrated, the AZGH 102 classified as lipophil compound and had suitable stability. This derivative absorbs and distributes faster in female than in male. The AUC 0-∞, absolute bioavailability, Cl and Vd were different in both sexes.

According to the obtained data, the AZGH 102 has a sex dependent pharmacokinetic in Wistar rats.

## Introduction

Nonsteroidal anti-inflammatory drugs (NSAIDs) such as naproxen, ibuprofen, diclofenac, are traditionally used for the treatment of pain, inflammation, and fever (1, 2). These drugs routinely exert their effects via inhibition of both cyclooygenases (COXs) isoforms. Recently, it is established that there are three COX isoenzymes, COX-1, COX-2, and COX-3 (2-4). COX-1 is responsible for cytoprotective effects and is important for maintenance of physiological functions of the human body (2, 4). COX-2 is an inducible isoenzyme which is involved in inflammatory processes (2, 4). COX-3 is similar to COX-1 and exerts antipyretic effect when inhibited by acetaminophen (3). The classic nonselective NSAIDs inhibit both COX-1 and COX-2, so they produce the desired effects such as anti-inflammatory as well as important side effects such as gastrointestinal disorders (2, 4). Moreover, it has been reported that inhibition of COX-2 reduces the risk of colorectal, breast, and lung cancers and slow the progress of Alzheimer’s disease (5). For these reasons researchers show interest in design and synthesis of selective COX-2 inhibitors. Coxibs such as celecoxib, rofecoxib, and valdecoxib are introduced as selective COX-2 inhibitors to the market but some of them such as rofecoxib and valdecoxib have been withdrawn from the market due to their adverse cardiovascular side effects (2, 4). 

Therefore, there is a need to develop new class of selective COX-2 inhibitors. Zarghi *et al.* reported that quinoline ring is a very suitable scaffold for COX-2 inhibitory activity and introduced the new derivatives of ketoprofen as novel class of selective COX-2 inhibitors in 2010 (1, 2). They reported 8-benzoyl-2-(4-(methylsulfonyl)phenyl)quinoline-4-carboxylic acid (AZGH 102) as a novel compound with similar IC50 to celecoxib besides improved selectivity index (COX-1/COX-2 inhibitory potency) in comparison with celecoxib (1). 

In this study the physicochemical properties of (AZGH 102) was entirely evaluated and the pharmacokinetic characteristics of this compound following intravenous (IV) and oral administration to male and female rats were investigated. 

## Materials and methods

*Materials*

**Figure 1:**
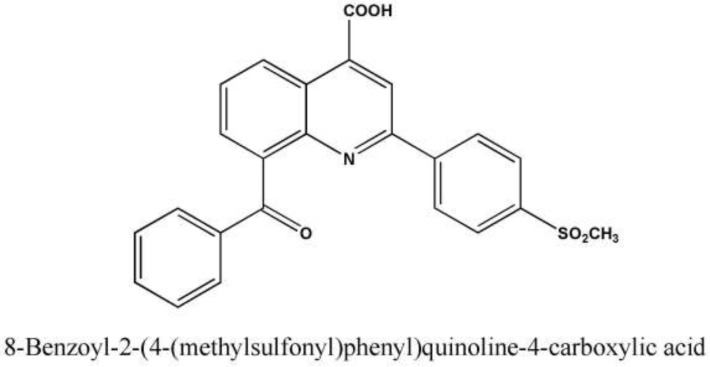
Chemical Structure of AZGH 102

The compound AZGH 102 (8-benzoyl-2-(4-(methylsulfonyl)phenyl)quinoline-4-carboxylic acid) (Figure 1) as novel derivative of ketoprofen was synthesized based on the previously reported method (1). Analytical grade acetonitrile and methanol were purchased from Merck (Darmstadt, Germany). Ultrapure water was obtained from Millipore Direct-Q system. All other chemicals and solvents were of analytical grade and provided from Merck (Darmstadt, Germany). Internal standards (IS) were obtained as a gift from the analytical laboratory of author’s institute.

*Structure and purity determination*

An Infra Red (IR) spectrum with KBr disk was acquired using a Termoelectron Co. Model Nicolet 380 spectrometer (USA). The mass spectral measurement was performed on a 6410 Agilent LCMS triple quadrupole mass spectrometer (LCMS) with an electrospray ionization (ESI) interface. A UV spectrum of the compound in the methanol was acquired by Shimadzu 1200 spectrometer (Japan) at a wavelength of 200-400 nm. Elemental analysis was performed for C and H. 

*HPLC Analysis*

*Apparatus and chromatographic conditions*

The Knauer chromatographic system was consisted of a Wellchrom 1001 pump, a Wellchrom K-2700 Diode Array Detector, a Wellchrom solvent degasser and a Rheodyne injector with 20 μL loop. Chromgate software version 3.1 was used for instrument control, data acquisition, and analysis. The separation of analyte was performed at ambient temperature (25 ºC) on the MZ C18 (250 × 4 mm, 5 µM) column from Merck (Darmstadt, Germany). The detector wavelength was fixed at 266 nm.

*Preparation of standard solutions and samples*

The working standard of AZGH 102 and ISs were prepared in methanol at 1 mg/mL concentration and stored at 2–8 ºC. 

Quality control (QC) samples were prepared at 20, 40, 80, 160, and 320 ng/mL concentrations by dilution of working standard in mobile phase.

Analytical samples were prepared by dilution of working standard in either mobile phase or spiking with blank plasma. 

*Analytical method development*

Method development was involved investigation of flow rates (1.5-2 mL/min) and mobile phase constitution. As a mobile phase, either acetonitrile or methanol in varying ratio (v/v) were added to 10 mM buffer phosphate. The effect of mobile phase pH (2-4) on peak resolution was also examined. 

*Plasma sample preparation*

Plasma samples were prepared by precipitating method and the efficacies of different precipitants were evaluated. In brief, the plasma samples were mixed with precipitants (acetonitrile, perchloric acid 12 and 24% and/or combination of NaOH 1 N and zinc sulphate 0.7 M) in different ratios and vortexed for 2 min. The obtained suspensions were centrifuged for 10 min at 10000 rpm and the supernatants were separated.

*Analytical method validation*

This method was validated for specificity, intra and inter-day precision, accuracy, limit of detection (LOD) and quantification (LOQ), linearity and stability according to ICH analytical method validation guideline.

The specificity was evaluated by comparison between blank samples and spiked ones with either AZGH 102 or IS. 

For linearity study, the calibration curves were constructed for AZGH 102 according to peak areas of five concentrations between 20 and 320 ng/mL with 5 replicates by linear regression, without weighting.

For intra-day precision, the relative standard deviation (RSD) of 3 replicates for quality control (QC) samples were calculated. The RSD of these samples were calculated in 3 replicates over 3 days to establish inter-day precision.

The difference between true value and measured concentration of 5 replicates for QC samples was used for accuracy determination.

The LOD was defined as the lowest concentration of analytes which produced response 3 times higher than background noise. The LOQ was also defined as the lowest concentration of analytes which could be determined with the RSD of 20% and accuracy within ± 20.

*Physicochemical properties*

Melting point was determined with an Electrothermal 9200 apparatus (UK). Moreover, aqueous solubility and octanol/water partition coefficient of the synthesized derivative was determined according to Organisation for Economic Co-operation and Development (OECD) Guideline for Testing of Chemicals, No.105 and No. 107, respectively (6-8).

The stability study was conducted according to forced degradation protocol ICH guideline for new drug substance (9-11). The study evaluated the stability of AZGH 102 at acidic, basic, and neutral medium. Moreover, the effect of oxidative condition (H_2_O_2_, 3 and 30%), thermal degradation at solid state and photolysis on the stability of AZGH 102 was evaluated. 

*Pharmacokinetic Studies*

*Animal experiments*

Male and female Wistar rats weighting 250 ± 10 g were used. They were housed in animal center of author’s institute. Each rat was housed in a cage which was kept at 25 ± 1 ºC temperature and controlled humidity with 12 h light/dark cycle. Animals were fasted overnight before the experiment and had free access to water. The study protocol was approved by the local ethics committee for animal experiments of Shahid Beheshti University of Medical Sciences (Tehran, Iran).

*Pharmacokinetic study*

The rats (12 male and 12 female) were randomly divided into four groups (6 rats/group) and assigned to receive AZGH 102 solution via intravenous (IV) and/or oral administration. The administered doses in both male and female groups for IV and oral studies were 10 mg/Kg and 20 mg/Kg, respectively. Blood samples were collected from the tail vein immediately prior to (blank sample) and at 0.08, 0.25, 0.5, 1, 2, 4, 6, 8, 10, 21 and 22 h after drug administration into heparinized micro-tubes. Blood samples were centrifuged at 1000 g for 10 min and the separated plasma samples were kept at – 20 ˚C until analysis. 

*Pharmacokinetic analysis*

Two-compartmental model was selected and pharmacokinetic analyses were performed using WinNonlin software (Pharsight Corporation, Mountain View, USA, Version 3.2). The following pharmacokinetic parameters were estimated: terminal elimination half-life (t1/2), area under the plasma concentration versus time curve from zero to the infinity (AUC 0-∞), distribution and elimination rate constants (α, β), y intercepts (A, B), volume of distribution (Vd), and total body clearance (CL). The peak plasma concentration (C_max_) and the time to reach C_max_ (T_max_) for oral dose were obtained directly from the observed individual plasma concentration-time data. 

*Statistical analysis*

Data were shown as the mean ± standard deviation (SD). Statistical analyses were performed using an unpaired t-test. A p-value of less than 0.05 was considered to be statistically significant.

## Results and Discussion

*Structure and purity determination*

**Figure 2:**
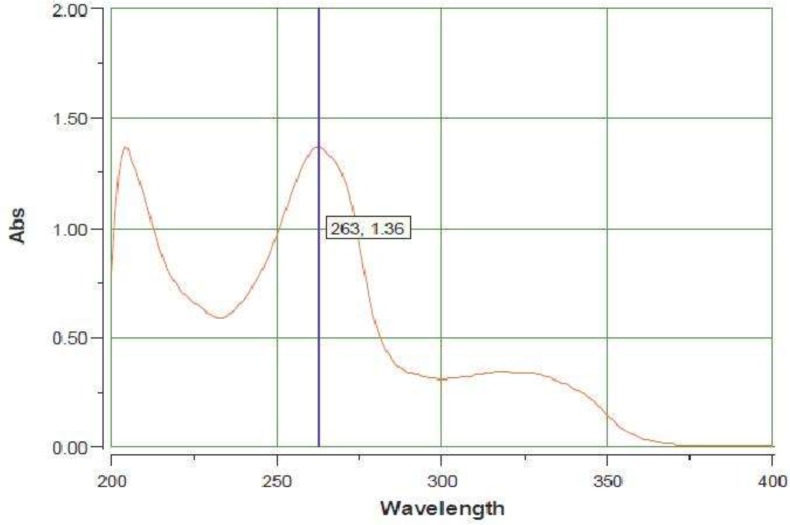
UV spectrum of AZGH 102 from 200-400 nm in methanol

The obtained result for IR spectrum of synthesized AZGH 102 was as follow: IR (KBr): ν (cm^-1^) 3158–2153 (OH), 1723, 1660 (C=O), 1281, 1150 (SO_2_). This result was similar to the reported one by Zarghi *et al.* for this compound (1). LCMS (ESI) chromatogram demonstrated a peak with 432.1 (M+1)^+^ which was in agreement with mass of AZGH 102 (Mw:431.08). The acquired data for elemental analysis were: Anal. Calcd for C_24_H_17_NO_5_S: C, 66.81; H, 3.97; N, 3.25. Found: C, 66.99; H, 3.80; N, 3.22 which was for C, N and H were within ± 0.4% of theoretical values. This data demonstrated the 99% purity for synthesized AZGH 102. The UV spectrum (Figure 2) demonstrated λmax at 266 nm.

**Figure 3:**
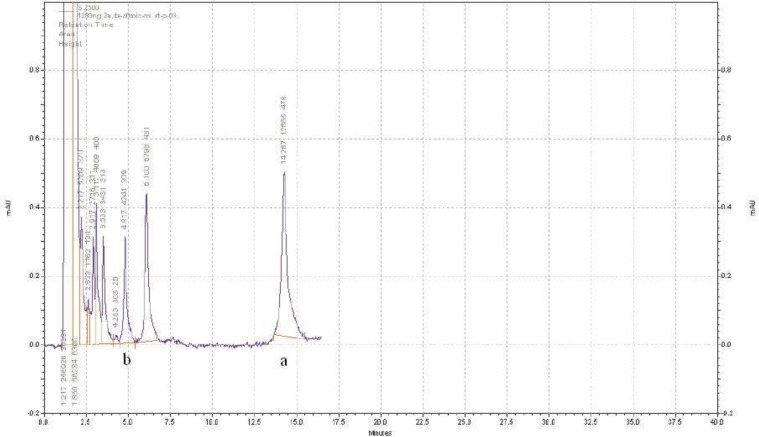
Sample HPLC chromatogram of AZGH 102 (b) and diclofenac as internal standard (a) in plasma. The separation was done with buffer phosphate (10 mM) at pH = 2.7 and acetonitrile (50:50 (v/v)) as mobile phase and the flow rate of 1.5 – 2 mL/min in gradient mode at time interval of 0 – 17 min

**Figure 4:**
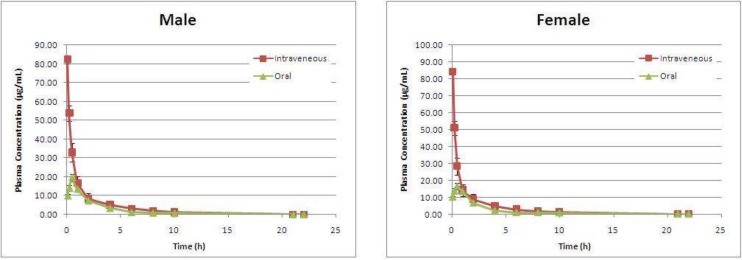
The plasma concentration versus time plots of AZGH 102 after IV (10 mg/Kg) and oral (20 mg/Kg) administrations in male and female Wistar rats

**Table 1:** HPLC validation parameters for AZGH 102 in methanol and plasma.

	Plasma	Methanol
Calibration range (ng/mL)	160-2560	20-320
Calibration points	5	5
Correlation coefficient (r)	0.9981	0.9985
Slope	5197	0.013
Intercept	-30.61	-5.410
Limit of quantification (LOQ) (ng/mL)	100	20
Limit of detection (LOD) (ng/mL)	40	7
Precision (RSD)a Intra (n=3)		
Level 1	0.15	0.08
Level 2	0.24	1.00
Level 3	3.06	4.86
Inter (n=9)		
Level 1	0.04	0.36
Level 2	0.48	0.21
Level 3	2.87	4.91
Accuracy (%)a		
Level 1	99.96	100.01
Level 2	100.02	100.02
Level 3	99.93	101.75

a: In plasma level 1 = 2560 ng/mL, level 2 = 640 ng/mL, level 3 = 160 ng/mL and in methanol , level 1 = 320 ng/mL, level 2 = 80ng/mL, level 3 = 20 ng/mL.

**Table 2:** Forced tests results for AZGH 102. The number of replication was 3.

**Stress Condition**	**Mean ±SD** a	**RSD** b
Hydrolysis(Acid)	0.94±0.02	2.13
Hydrolysis(Base)	0.92±0.01	1.08
Hydrolysis (Buffer pH=7)	0.95±0.04	4.21
Oxidative stress (H2O2-3%)	0.94±0.02	2.13
Oxidative stress (H2O2-30%)	0.90±0.04	4.45
Thermal Degradation (solid phase)	0.95±0.03	3.16
Photodegradation (Acid)	0.93±0.02	2.15
Photodegradation (Base)	0.96±0.04	4.16
Photodegradation (Buffer pH=7)	0.93±0.02	2.15

a : Standard deviation

b : Relative standard deviation

**Table 3:** The calculated pharmacokinetic parameters (mean ± SD) for AZGH 102 in oral (20 mg/Kg) and intravenous (10 mg/Kg) administration to Wistar rats

		**Oral route**			**Intravenous route**	
	**Female**	**Male**	**P value**	**Female**	**Male**	**P value**
β (h-1)	0.19 ± 0.00	0.19 ± 0.01	NS	0.54 ± 0.01	0.29 ± 0.01	NS
Ka (h-1)	1.26 ± 0.34	0.69 ± 0.15	0.002	-	-	-
α (h-1)	3.70 ± 1.56	5.79 ± 2.33	0.049	3.87 ± 1.11	2.98 ± 0.67	0.029
t1/2 Ka (h)	0.59 ± 0.15	1.04 ± 0.20	0.001	-	-	-
t1/2 β (h)	3.72 ± 0.10	3.71 ± 0.16	NS	2.38 ± 0.12	2.23 ± 0.74	NS
t1/2 α (h)	0.21 ± 0.06	0.14 ± 0.05	0.026	0.19 ± 0.05	0.24 ± 0.05	0.027
Tmax (h)	0.50 ± 0.00	0.50 ± 0.00	NS	-	-	-
Cmax (μg/mL)	16.49 ± 1.05	19.64 ± 1.96	0.003	-	-	-
AUC 0-∞ (h.μg/mL)	43.92 ± 0.71	49.33 ± 3.38	0.002	85.75 ± 9.23	91.33 ± 17.13	NS
Vd (mL)	156.27 ± 4.54	147.04 ± 9.43	0.028	101.26 ± 13.73	99.73 ± 15.21	NS
Cl (mL/h)	29.15 ± 0.47	27.47± 1.81	0.026	29.46 ± 3.19	28.20 ± 5.02	NS
F (%)	25.59± 0.40	27.00± 1.85	0.049	-	-	-
C0 (μg/mL)	-	-	-	100.47 ± 14.08	113.45 ± 7.99	0.014

*Analytical method development*

The retention time and area under the curve (AUC) of analytes peaks were affected by the flow rate changes. When the flow rate increased, the AUC of analytes peaks decreased. This effect was in accordance with Chen *et al.* report (12). The appropriate flow rate was chosen according to the peaks resolution and retention time of the analyte. Substituting acetonitrile with methanol resulted in reducing the analytes retention times and column pressure as well as increasing the peak sharpness and resolution. 

The pH of mobile phase especially at the range of 2.5-3.5 affected the retention time and peak sharpness as well. The results showed that the mixture of buffer phosphate (10 mM) at pH = 2.7 and acetonitrile (50:50 (v/v)), with the flow rate of 1.5 -2 mL/min in gradient mode at time interval of 0 – 17 min, can separate AZGH 102 from interfering peaks properly. Among proposed compounds as an IS (Celecoxib, piroxicam, meloxicam, naproxen, ibuprofen and diclofenac); only diclofenac (50 ng/mL) had a suitable retention time of 15 min (Figure 3).

*Analytical method validation*

There were no interfering peaks in the chromatogram of blank (methanol), spiked samples with plasma and blank plasma.

The obtained results for precision, linearity, accuracy, LOD and LOQ of AZGH 102 in methanol and plasma are presented in Table 1. Since the obtained RSD in all concentrations were less than 5%, this method had a suitable precision. The accuracy was in the suitable range, also. 

The obtained LOD and LOQ for AZGH 102 at methanolic medium was 7 and 20 ng/mL, respectively. Whereas the obtained LOD and LOQ for AZGH 102 at plasma medium was about 4 times greater than methanolic medium and about 40 and 100 ng/mL, respectively. 

All calibration curves showed good linearity (r^2 ^> 998) in the selected range either in methanol (20-320 ng/mL) or plasma (160-2560 ng/mL). The obtained slope and intercept for AZGH 102 in methanol was 0.013 and -5.41, respectively. These parameters for AZGH 102 in plasma were 5197 and -30.61. The difference between the slopes and the zero were significant (P < 0.05). Moreover, the intercepts showed no significant difference with zero (P > 0.05).

Recovery study demonstrated that AZGH 102 can be recovered up to 73.1 ± 2.0 % at the optimum condition. The recovery percent for diclofenac at the similar condition was 72.5 ± 2.1.

*Physicochemical properties*

The melting point of AZGH 102 was 228 °C which was in the reported range by Zarghi et al. for this compound (1). The obtained aqueous solubility for AZGH 102 was 2.7 ± 0.09 (mg/L) for 3 replications. The solubility of ketoprofen and celecoxib as selective COX-2 inhibitor in water was 51 and 3.3 (mg/L), respectively. This new derivative has water solubility similar to celecoxib. The log P parameter or logarithm of octanol/water partition coefficient which demonstrated the lipophilicity of compounds was 3.75 ± 0.001 for 3 replications. Aqueous solubility and log P parameters demonstrated that AZGH 102 can be considered as a lipophilic compound. AZGH 102 was more lipophil than celecoxib and ketoprofen with log P of 3.4 and 3.1 respectively (13, 14). According to the obtained log P and water solubility for AZGH 102, this new derivative of ketoprofen has similar physicochemical properties to celecoxib which is known as potent COX-2 inhibitor and has suitable physico-chemical properties according to Lipinskiʹs rule of five (15).

The stability study results were shown in the Table 2. As shown, in all of the examined conditions more than 90% of AZGH 102 was recovered intact. This percent of recovery demonstrate the suitable stability of AZGH 102.

*Pharmacokinetic study*

The plasma concentration versus time plots of AZGH 102 after IV (10 mg/Kg) and oral (20 mg/Kg) administrations in male and female rats have been shown in Figure 4. 

Concentration-time data for IV and oral administration were best fitted with two compartment model.The calculated pharmacokinetic parameters of AZGH 102 in both sexes have been demonstrated in Table 3.

*Pharmacokinetics of AZGH 102 in rat following intravenous administration*

As the data shown in Table 3, the elimination rate constant (β) and distribution rate constant (α) in male group were 0.29 ± 0.01 and 2.98 ± 0.67 (h-1), respectively. In female group the β_female_ (0.54 ± 0.01 h-1) showed no significant differences with male group (P > 0.05) while α_female_ (3.87 ± 1.11 h-1) demonstrated significant differences with male group (P = 0.029). 

Apparent volume of distribution (Vd) which affected the total body clearance of the drug showed no differences between both sexes at IV route (101.26 ± 13.73 mL for female and 99.73 ± 15.21 mL for male).

At IV route the Cl dose not affected by sex (Cl_male_ = 28.20 ± 5.02 mL/h vs. Cl_female_ = 29.46 ± 3.19 mL/h).

*Pharmacokinetics of AZGH 102 in rat following Oral administration*

The elimination rate constant (β) and distribution rate constant (α) in male group were 0.19 ± 0.01 and 5.79 ± 2.33 (h-1) respectively. In female group, the β_female_ (0.19 ± 0.00 h-1) showed no significant differences with male group (P > 0.05) while α_female_ (3.70 ± 1.56 h-1) demonstrated significant differences with male group (P < 0.05). 

At oral route the AZGH 102 was absorbed faster in female than in male rats (Ka = 1.26 ± 0.34 versus 0.69 ± 0.15 h-1) while was distributed slower in female than in male. At IV route different phenomenon was seen, the distribution phase was faster in female than in male. It can be proposed that at oral route in female rats due to faster absorption phase, the enzymes or mechanisms involved in the distribution process was saturated and the rate of distribution decreased. The observed Cmax which was about 20% lesser in female than in male (C _max, male_ = 19.64 ± 1.96 μg/mL vs. C _max, female_ = 16.49 ± 1.05 μg/mL) may support this hypothesis. 

At oral route Vd varied between sexes (147.04 ± 9.43 mL for male and 156.27 ± 4.54 mL for female) with P = 0.028. At both routes of drug administration the female sex demonstrated larger Vd; so, the drug clearance would have been different in both sex. As the data showed in Table 3, the clearance of AZGH 102 was significantly (P = 0.026) grater (about 7%) in female at oral route (Cl_male_ = 27.47 ± 1.81 mL/h vs. Cl_female_ = 29.15 ± 0.74 mL/h). 

Due to difference in volume of distribution and clearance between male and female ,at oral route it is expected the AUC 0-∞ demonstrated significant differences in both sexes. The obtained data for AUC 0-∞ confirm the obtained data for Vd and Cl. As data showed in Table 3, AUC was about 10% (P = 0.002) smaller in female group (43.92 ± 0.71 h.μg/mL vs. 49.33 ± 3.38 h.μg/mL). However, the Cl, Vd and AUC 0-∞ showed no significant differences at IV route between male and female rats.

The absolute bioavailability for female and male was 25.59 ± 0.04 % and 27.00 ± 1.85 %, respectively. This compound showed sex difference in bioavailability (P = 0.049).

## Conclusion

The new synthesized derivative of ketoprofen (AZGH 102) cause ketoprofen obtained similar physicochemical properties to celecoxcib. AZGH 102 demonstrates suitable stability according to forced degradation protocol ICH guideline for new drug substance. This derivative absorbs and distributes faster in female than in male. The distribution phase is saturable. However, the elimination rate is similar in both sexes at IV and oral route. It seems that due to differences between sexes in absorption and distribution phase of AZGH 102 at oral route the AUC 0-∞, Cl and Vd were different in both sexes.

## References

[1] Zarghi A, Ghodsi R (2010). Design, synthesis, and biological evaluation of ketoprofen analogs as potent cyclooxygenase-2 inhibitors. Bioorg. Med. Chem..

[2] Zarghi A, Ghodsi R, Azizi E, Daraie B, Hedayati M, Dadrass OG (2009). Synthesis and biological evaluation of new 4-carboxyl quinoline derivatives as cyclooxygenase-2 inhibitors. Bioorg. Med. Chem..

[3] Chandrasekharan NV, Dai H, Roos KL, Evanson NK, Tomsik J, Elton TS, Simmons DL (2002). COX-3, a cyclooxygenase-1 variant inhibited by acetaminophen and other analgesic/antipyretic drugs: cloning, structure, and expression. Proc. Natl. Acad. Sci. U S A..

[4] Kim KJ, Choi MJ, Shin JS, Kim M, Choi HE, Kang SM, Jin JH, Lee KT, Lee JY (2014). Synthesis, biological evaluation, and docking analysis of a novel family of 1-methyl-1H-pyrrole-2,5-diones as highly potent and selective cyclooxygenase-2 (COX-2) inhibitors. Bioorg. Med. Chem. Lett..

[5] Zarghi A, Najafnia L, Daraee B, Dadrass OG, Hedayati M (2007). Synthesis of 2,3-diaryl-1,3-thiazolidine-4-one derivatives as selective cyclooxygenase (COX-2) inhibitors. Bioorg. Med. Chem. Lett..

[6] OECD (1989). Guideline for Testing of Chemicals, no.117: Partition Coefficient (n-octanol/water), High Performance Liquid Chromatography Method.

[7] OECD (1995). Guideline for Testing of Chemicals, no.107: Partition Coefficient (n-octanol/water), Shake Flask Method.

[8] OECD (1995). Guideline for Testing of Chemicals, no.105: Water Solubility.

[9] International Conference on Harmonization (1999). ICH Specification: Test Procedures and Acceptance Criteria For New Drug Substances and New Drug Products (Q6A).

[10] International Conference on Harmonization (1999). ICH, Impurities: Impurities in New Drug Substances (Q3A).

[11] Bakshi M, Singh B, Singh A, Singh S (2001). The ICH guidance in practice: stress degradation studies on ornidazole and development of a validated stability-indicating assay. J. Pharm. Biomed. Anal..

[12] Chen CL, Uckun FM (2000). Highly sensitive liquid chromatography-electrospray mass spectrometry (LC-MS) method for the determination of etoposide levels in human serum and plasma. J. Chromatogr. B Biomed. Sci. Appl..

[13] National Center for Biotechnology Information (2015). PubChem Compound Database; CID=3825. Ketoprofen.

[14] National Center for Biotechnology Information (2015). PubChem Compound Database; CID=2662. Celecoxib.

[15] Leeson P (2012). Drug discovery: Chemical beauty contest. Nature..

